# Multimodal Ligand Binding Studies of Human and Mouse G-Coupled Taste Receptors to Correlate Their Species-Specific Sweetness Tasting Properties

**DOI:** 10.3390/molecules23102531

**Published:** 2018-10-03

**Authors:** Fariba M. Assadi-Porter, James Radek, Hongyu Rao, Marco Tonelli

**Affiliations:** 1 Department of Biochemistry, University of Wisconsin-Madison, Madison, WI 53706, USA; jtradek@yahoo.com (J.R.); hrao@wisc.edu (H.R.); tonelli@nmrfam.wisc.edu (M.T.); 2 National Magnetic Resonance Facility at Madison, University of Wisconsin-Madison, Madison, WI 53706, USA; 3 Department of Integrative Biology, University of Wisconsin-Madison, Madison, WI 53706, USA

**Keywords:** G-coupled protein receptors (GPCRs), sweet taste receptor, ligand binding, nuclear magnetic resonance spectroscopy (NMR), saturation transfer difference (STD)-NMR, differential scanning calorimetry (DSC), circular dichroism (CD) spectroscopy, intrinsic fluorescence spectroscopy (IF)

## Abstract

Taste signaling is a complex process that is linked to obesity and its associated metabolic syndromes. The sweet taste is mediated through a heterodimeric G protein coupled receptor (GPCR) in a species-specific manner and at multi-tissue specific levels. The sweet receptor recognizes a large number of ligands with structural and functional diversities to modulate different amplitudes of downstream signaling pathway(s). The human sweet-taste receptor has been extremely difficult to study by biophysical methods due to the difficulty in producing large homogeneous quantities of the taste-receptor protein and the lack of reliable in vitro assays to precisely measure productive ligand binding modes that lead to activation of the receptor protein. We report here a multimodal high throughput assay to monitor ligand binding, receptor stability and conformational changes to model the molecular ligand-receptor interactions. We applied saturation transfer difference nuclear magnetic resonance spectroscopy (STD-NMR) complemented by differential scanning calorimetry (DSC), circular dichroism (CD) spectroscopy, and intrinsic fluorescence spectroscopy (IF) to characterize binding interactions. Our method using complementary NMR and biophysical analysis is advantageous to study the mechanism of ligand binding and signaling processes in other GPCRs.

## 1. Introduction

The human sweet taste receptor is a heterodimeric complex of the proteins T1R2 and T1R3. The complex is a member of the G-protein-coupled receptor class (GPCR); which share a common design of a seven transmembrane heptahelical domain with an extracellular N-terminus and intracellular C-terminus. The downstream signaling pathway of the receptor is thought to be controlled by G-proteins that stimulate the synthesis of intracellular second messengers such as cyclic AMP, inositol phosphate and Ca^2+^ ions. The sweet GPCR has been further grouped as a class C receptor, of which there are 4 other families: class A rhodopsin family, class B secretin family, class D adhesion family and class E frizzles/smoothened family. The class C GPCR share a common structure of a large amino-terminal domain (ATD), which serves as the principle ligand-binding domain, followed by a short cysteine-rich domain (CRD) tied to the transmembrane domain (TMD) and intracellular C-terminal domain [1,2]. 

**Figure 1:**
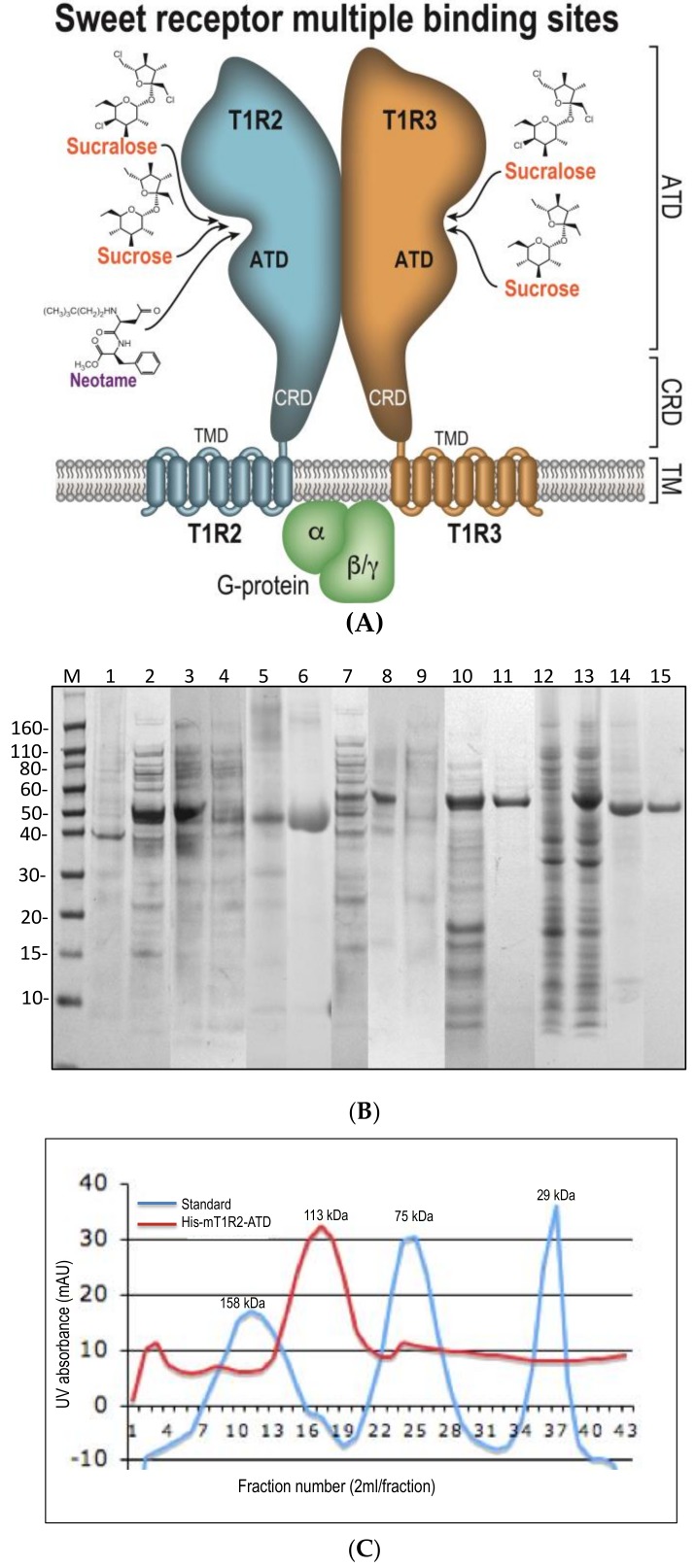
(**A**) Model of sweet receptor and its proposed interaction sites with sweet ligands. (**B**) SDS-PAGE of the expression and purification of human and mouse proteins. Lanes shown are: M. Novex Sharp Pertained Protein Standard (kDa); 1. −IPTG, total proteins; 2. +IPTG, total cell proteins containing His-TEV-ATD-hT1R2 (~56 kDa); 3. Pellet: His-ATD-hT1R2; 4. Supernatant: His-ATD-hT1R2; 5. Refolded His-ATD-hT1R2; 6. FPLC purified His-ATD-hT1R2; 7. +IPTG, total proteins, His-TEV-ATD-mT1R2 (~63 kDa); 8. Pellet of His-ATD-mT1R2+IPTG; 9. Supernatant of His-ATD-mT1R2; 10. Refolded His-ATD-mR2; 11. Purified His-ATD-mT1R2, by FPLC; 12. −IPTG; 13. +IPTG, His-Sumo-TEV-ATD-hT1R2; 14. Cut His-Sumo-TEV-ATD-hR2 (ATD-hT1R2, ~54 kDa and His-Sumo-TEV, ~12.5 kDa); 15. FPLC purified ATD-hT1R2. (**C**) Tev protease cleaved ATD protein peaks off the Superdex 200 prep grade FPLC column.

The sweet-taste receptor has been shown to bind a large ensemble of molecules such as sugars, artificial sweeteners, sweet-tasting proteins and some D-amino acids that mediate the sweet taste response (Figure 1A). Regions of the complex that bind specific ligands include the ATD of human (h) T1R2: non-caloric sweeteners aspartame, neotame, sucralose and monellin, a sweet-tasting protein; the ATD of T1R3: cyclamate, neohesperidin dihydrochalcone and lactisole; and the ATD and CRD of hT1R3: sweet-tasting proteins brazzein and neoculin [3,4,5,6,7]. T1R2 and T1R3 subunits have been shown to bind natural sugars glucose and sucrose with distinct affinities even though the individual contributions of each subunit to the interaction are unknown.

One of the major difficulties in studying the molecular details of the function of this complex has been the lack of a reliable method for producing large quantities of purified proteins using recombinant technology. The mouse (m) versions of T1R2 and T1R3 ATDs have been successfully produced, but only in small quantities and as fusion proteins [8]. Recently, hT1R3 ATD was purified and characterized [9]. Here, we describe multi-modal screening methodologies that are required for the complex heterodimeric sweet taste receptor. First, we will describe a method for producing highly purified protein constructs for both human and mouse fusion small ubiquitin-like modifier (SUMO)-T1R2 ATD protein and the protease cut T1R2 ATD protein [10]. Gel filtration chromatography demonstrated that the T1R2 ATD exists in a dimeric configuration. Second, we will describe several complementary methods for the study of the constructs and their interaction with small ligands that elicit a sweet-taste response in tests conducted in vivo with human taste panels [11] and in vitro by heterologous calcium assays with HEK211 cells overexpressing the sweet taste receptor [12]. Using circular dichroism spectroscopy, we show that there is a decrease in the overall α-helical content of the construct upon binding to sweet ligands. In addition, there appears to be an overall decrease in thermal stability in the tertiary structure of the SUMO-hT1R2 ATD fusion protein upon binding of neotame, a small sweet-taste inducing dipeptide ligand. Saturation transfer difference spectra confirmed that molecules eliciting a CD response also gave positive difference spectra indicating binding of ligands to the ATD domain. Furthermore, we show that combination of intrinsic fluorescence spectroscopy, circular dichroism spectroscopy and saturation transfer difference spectroscopy can be useful in evaluating detailed molecular changes at the receptor level while monitoring ligand binding. These complementary methods provide important tools for studying allosteric effects of one sweet-taste responding molecule over another, as described by the heterologous calcium assay for sweet-taste response [12]. In this work, we demonstrate that we can study the highly purified sweet-taste protein and its binding of target molecules using these biophysical methods. 

## 2. Results

We report here the results of experiments using multimodal biophysical techniques that probe the interaction of human and mouse T1R2 ATD with small ligands that are known to elicit a sweet-taste response. The production and purification of functional sweet-taste receptor proteins has been a challenge due to its large size and number of cysteines that need to be oxidized in the right form in order for the receptor to be functionally active. 

### 2.1. Protein Production and Purification of the Human and the Mouse ATD T1R2 Proteins

We report here the successful construction and conditions that allow for the study of functionally relevant proteins. The constructs used in this study were from extracellular amino terminal domains (ATD) of the sweet taste receptor and contain ligand-binding sites (Figure 1A). The ATD region of the protein was cloned into both a 8xHis tag vector with a tobacco etch virus protease (TEV) cleavage site and a recombinant fragment of ULP1 (Ubl-specific protease 1) from Saccharomyces cerevisiae (SUMO) vector. The proteins were purified to near homogeneity as described by the 12% SDS-PAGE profiles (Figure 1B). The protein from both constructs was found to be in the form of a homodimer as determined by comparison to molecular weight standards in a gel filtration column (Figure 1C). 

### 2.2. Ligand Binding to the Human Receptor T1R2 ATD Results in Secondary Structural Changes

**Figure 2:**
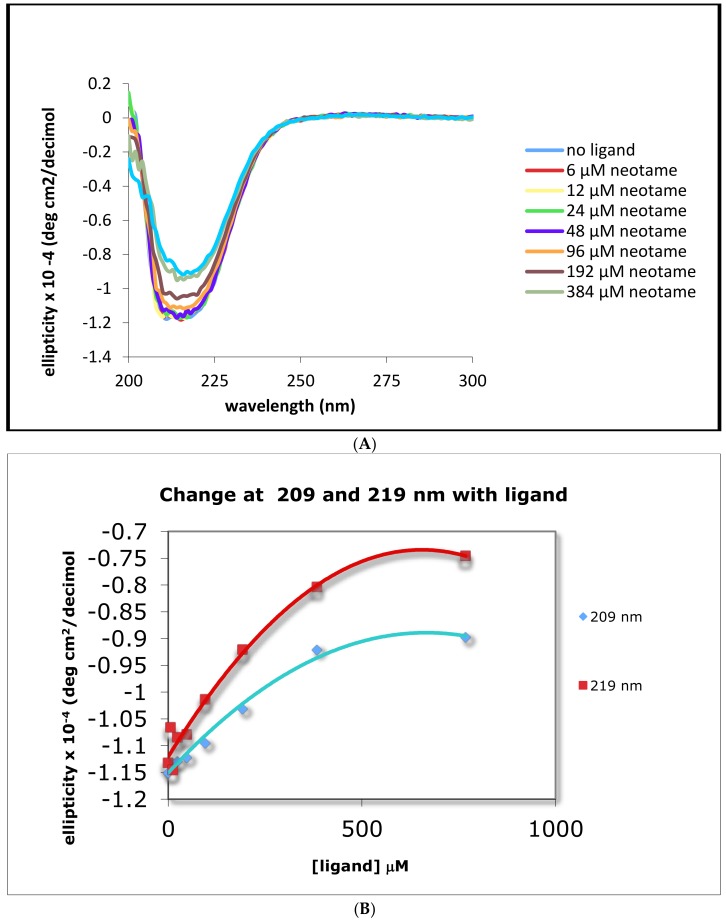

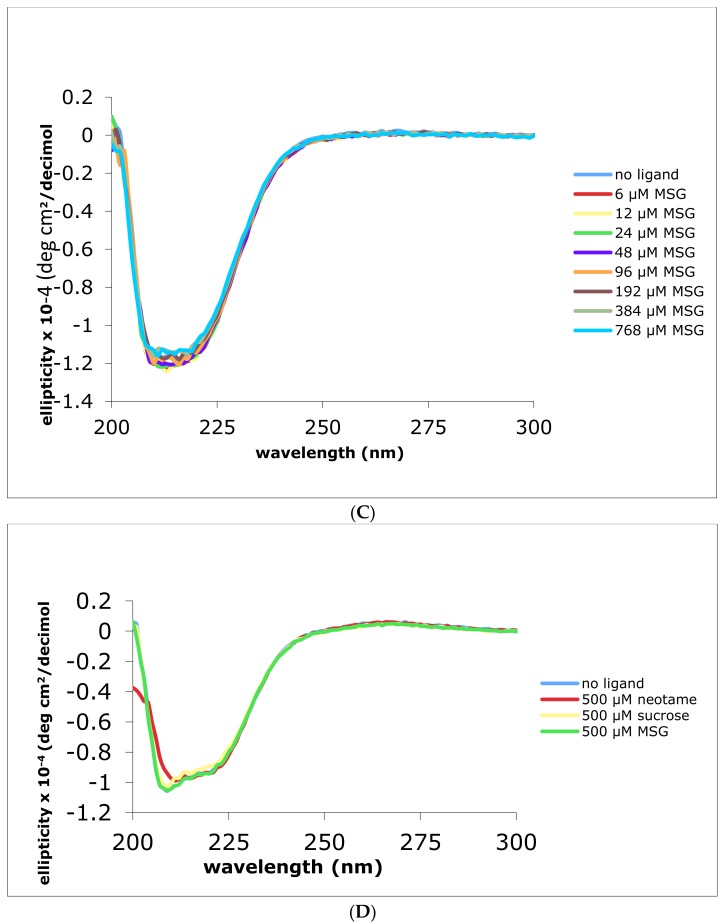
CD spectra of His tagged human and mouse T1R2 ATD ± ligands. Panel (**A**) human T1R2 ATD ± neotame, Panel (**B**) changes in ellipticity at 209 and 219 nm with increasing concentrations of neotame, black symbols are actual data and colored symbols are polynomial-fitted values; panel (**C**) human T1R2 ATD ± MSG; panel (**D**) mouse T1R2 ATD with addition of saturating concentrations of neotame, sucrose, or MSG.

We used circular dichroism (CD) to examine changes in the ATD secondary structure after addition of relevant ligands concentrations to the human and the mouse ATDs. There was a small but measurable increase in the overall molar ellipticity values upon addition of neotame (a synthetic dipeptide and the human specific binding ligand) resulting in a loss of about 5% in the α-helix content of the protein. Neotame showed binding to the His-hT1R2 ATD as demonstrated by CD (Figure 2A). The changes in ellipticity at 209 and 219 nm that were observed in a titration study with increasing concentrations of neotame are shown in Figure 2B. As a negative control, we used the non-sweet monosodium glutamate (MSG). As expected, MSG produced no change in the CD profiles especially at 209 and 219 nm (Figure 2C). A summary of calculated dissociation binding constant (*K*_D_) values for other sweeteners is reported in Table 1 for the human T1R2 ATD. 

Neotame is thought to bind only to human T1R2 ATD, thus we next examined the CD properties of the mouse analogue in response to addition of neotame. Figure 2D demonstrates that when mouse His-T1R2 ATD was substituted for the human counterpart, addition of neotame up to the highest concentration (500 μM), had no effect on the loss of ellipticity as compared to the corresponding human analogue (compare Figure 2A,D). Sucrose that commonly binds to both mouse and human sweet receptors showed a small decrease in ellipticity upon binding to these ATDs. As expected, the negative control, MSG, showed no change in ellipticity in the mouse ATD. 

### 2.3. Intrinsic Fluorescence Measurements Indicate That Ligand Binding Affects Tryptophan Residues in Human and Mouse T1R2 ATDs

**Figure 3:**
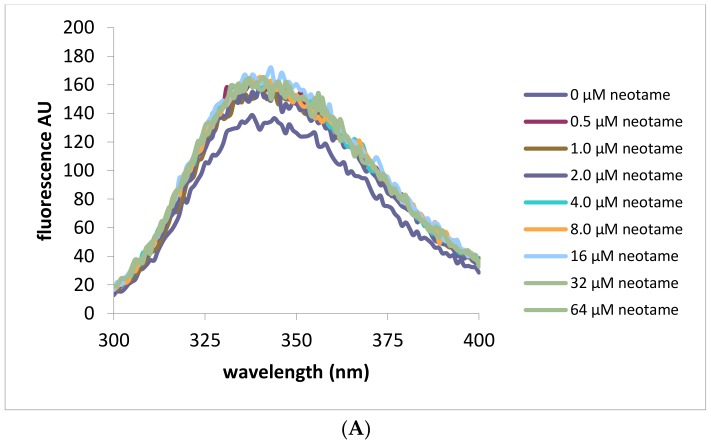

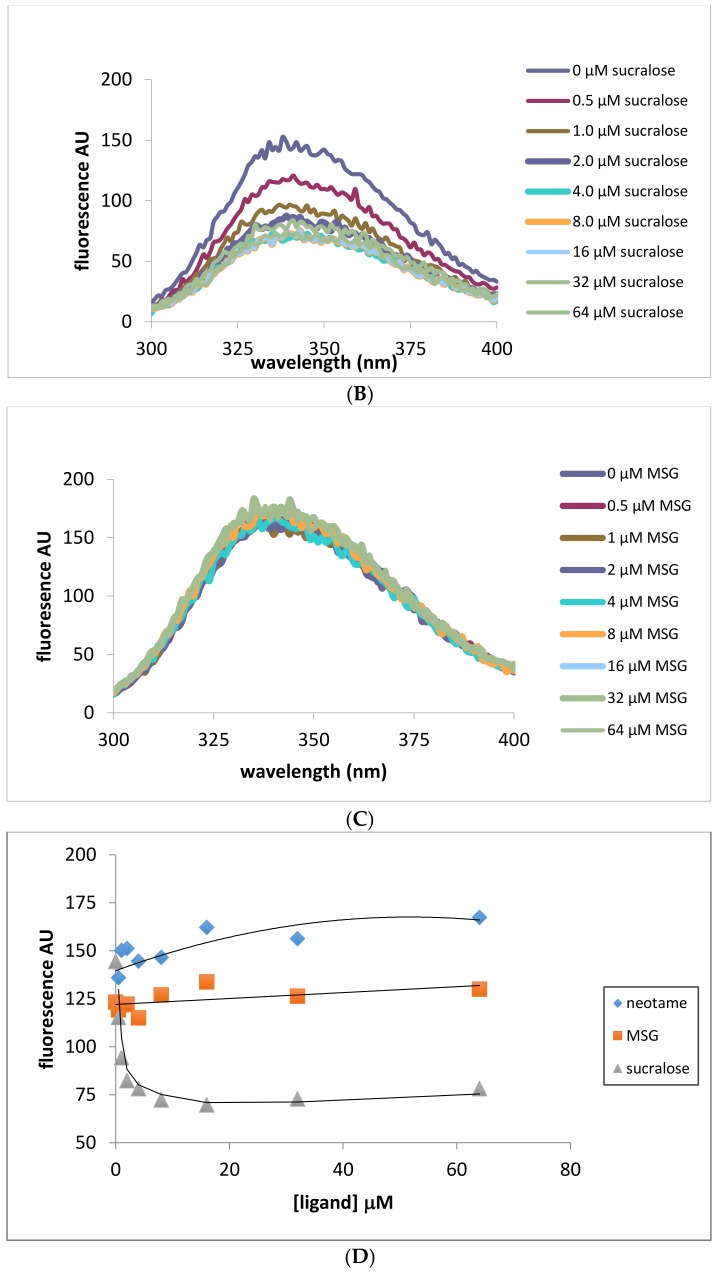

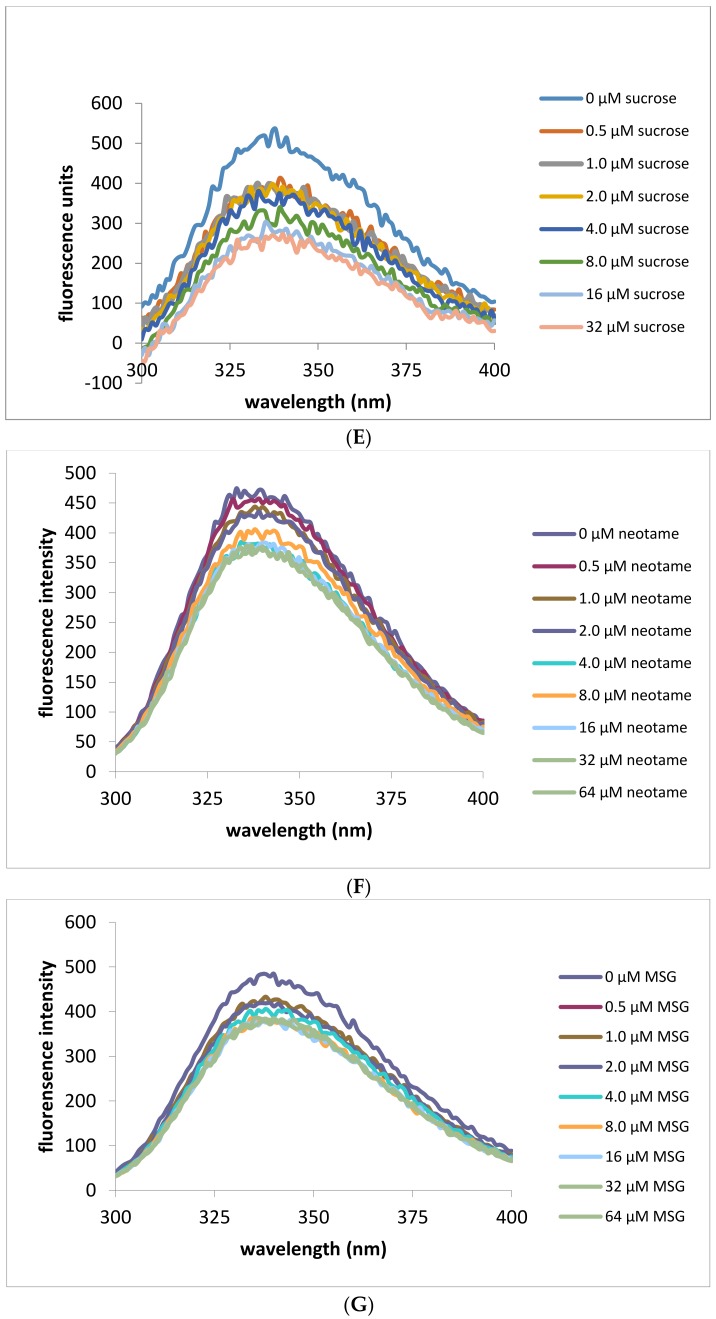

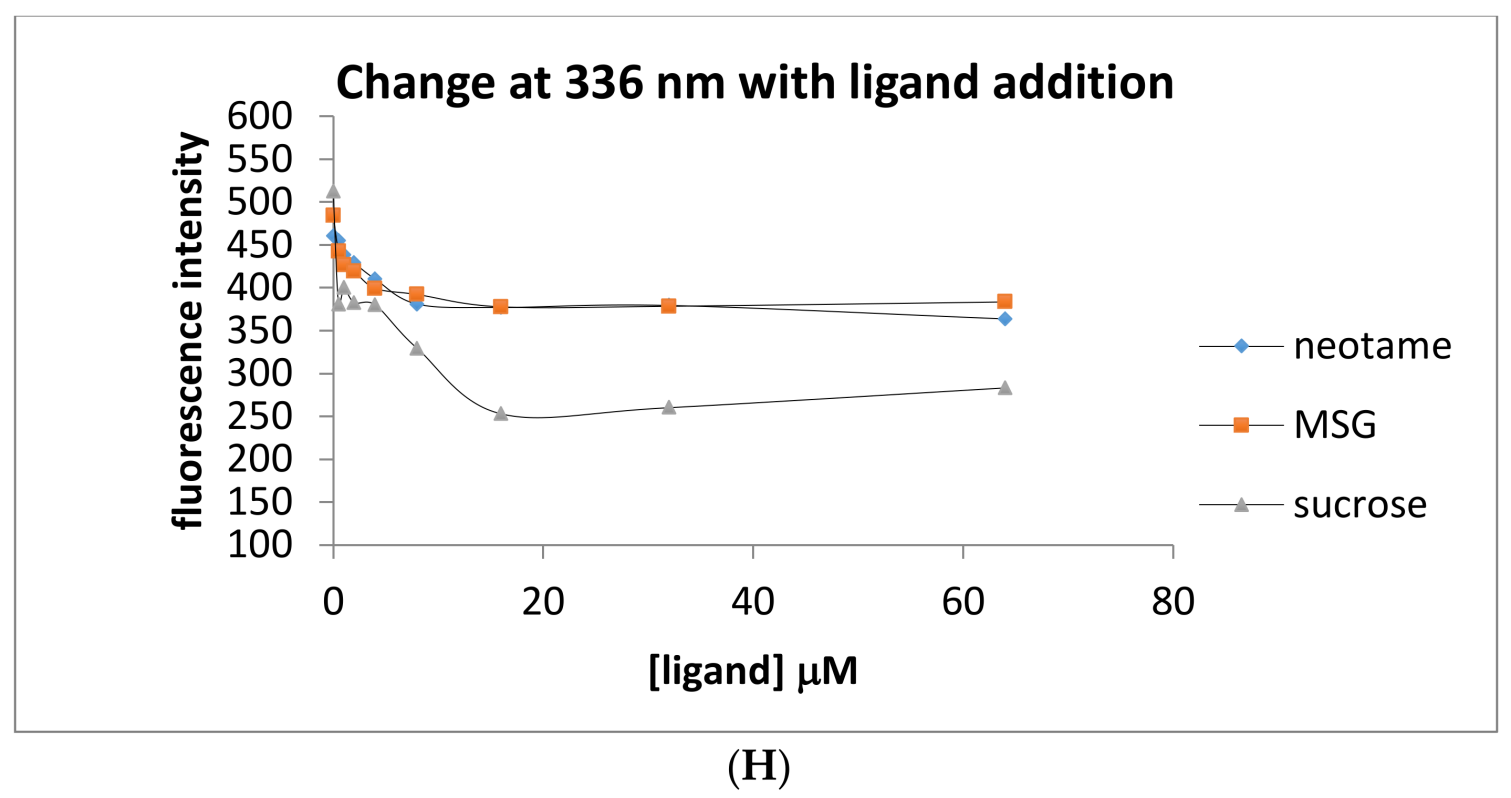
Intrinsic fluorescene emission spectra (excitation at 280 nm) of His tagged human and mouse T1R2 ATD. Panel (**A**) human T1R2 ATD ± neotame; (**B**) human T1R2 ATD ± sucralose; (**C**) human T1R2 ATD ± MSG; (**D**) changes in intrinsic fluorescence signal at 336 nm in the presence of human T1R2 ATD and increasing concentrations of either neotame, sucralose or MSG; (**E**) mouse T1R2 ATD ± sucrose; (**F**) mouse T1R2 ATD ± neotame; (**G**) mouse T1R2 ATD ± MSG; (**H**) changes in intrinsic fluorescence signal at 336 nm in the presence of mouse T1R2 ATD and increasing concentrations of either sucrose, neotame, or MSG.

Intrinsic fluorescence measurements were also performed in order to exploit the changing environment around tryptophan residues as a marker for ligand binding. Addition of neotame in increasing concentrations (0–64 μM) was monitored by measurements of intrinsic fluorescence of tryptophan residues as shown in Figure 3. Fluorescence intensity was found to increase upon addition of neotame to the HIS hT1R2 ATD with a maximal value occurring at 10 μM (Figure 3C). Interestingly, adding sucralose produced the opposite effect on tryptophan residues by decreasing fluorescence intensity (Figure 3B,D), suggesting different modes of binding interactions for these ligands. In addition, sucralose in the h-T1R2 ATD reached half of the maximal value at half the concentration of neotame (5 μM). On the other hand, MSG produced no change in intrinsic fluorescence intensity over the same concentration range as the other ligands. It should be noted that sucrose (natural analog of sucralose) did not change the fluorescence intensity despite its sweet response in other studies such as calcium imaging assay and taste studies. When the mouse ATD protein was used (Figure 3E–H), neotame and MSG did not change the intrinsic fluorescence intensity, while sucrose produced a decrease in the fluorescence intensity with a half-maximal value of 8 μM (Figure 3H). These results are in accordance with the fact that neotame elicits a sweet-taste response in the human, but not in the mouse analogue. 

### 2.4. Monitoring Direct Ligand Binding by Saturation Transfer Difference (STD) NMR Spectroscopy

**Figure 4:**
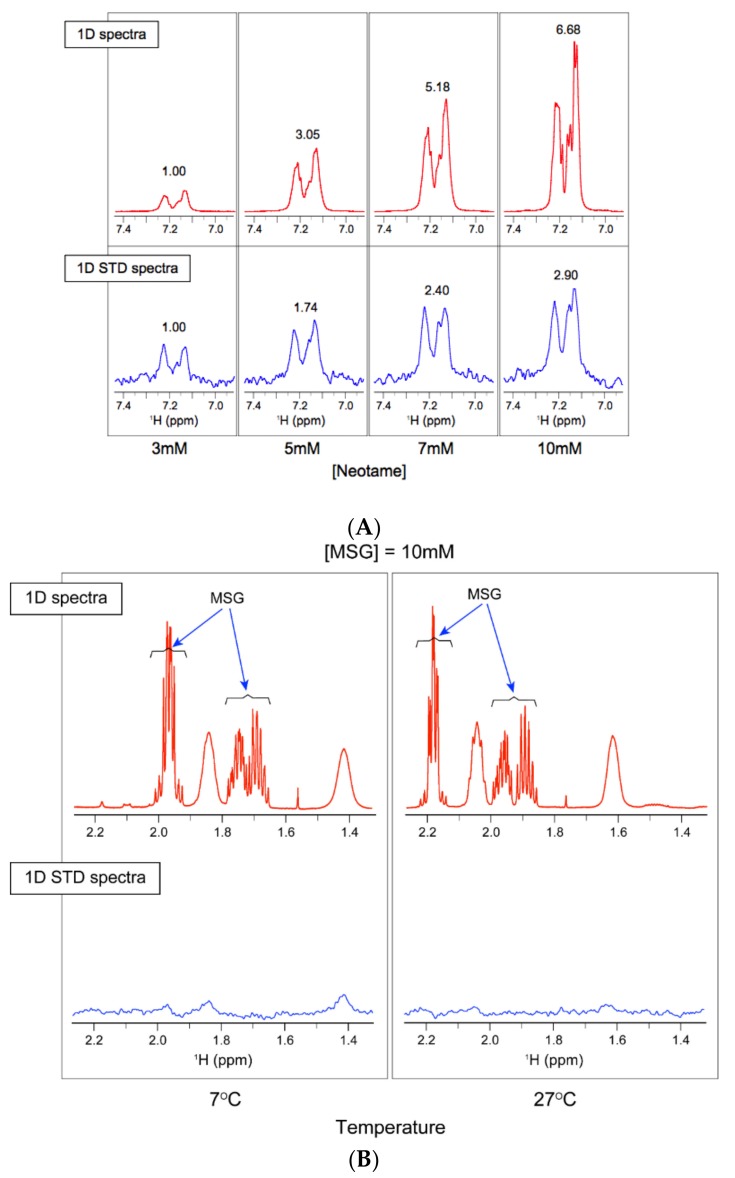
Saturation transfer difference spectra of His tagged human T1R2 ATD. NMR data were collected on a Varian VNMRS spectrometer operating at 800 MHz and equipped with a cryogenic probe. (**A**) Human T1R2 ATD ± neotame at 27 °C. Top panel shows one-dimensional (1D) ^1^H-NMR, and the bottom panel shows 1D STD NMR spectra of neotame at increasing concentrations (3–10 mM). The observed STD signals increase with the concentration of neotame, indicating that neotame binds to the receptor domain; (**B**) human T1R2 ATD ± MSG (negative control) measured at 7 and 27 °C. Top panel shows 1D ^1^H-NMR with MSG proton signals, and bottom panel shows STD spectra. Lack of STD signal in MSG spectra indicates no binding.

We applied STD-NMR spectroscopy as a sensitive assay to monitor binding between the correctly folded T1R2 ATD and a ligand. We used this approach to investigate the interaction between human ATD and the sweet ligand neotame (Figure 4A). This assay allowed us to optimize the STD response by varying the neotame concentration at a given receptor concentration. The STD spectrum from neotame binding to the human extra cellular domain yielded proton NMR signals that arise from specific interactions with the h-T1R2 ATD, as shown in the bottom panel of Figure 4A. However the range of concentrations of neotame eliciting response appears to be higher than the reported measured *K*_D_ by CD reported in Table 1. This could be due to lower sensitivity of the NMR technique as compared to CD. To distinguish between STD signals from functional ATD interactions and non-specific interactions, we also utilized MSG as a negative control (Figure 4B). MSG was chosen because it is known to only interact with the T1R1 subunit of the umami receptor but not with the sweet receptor. Accordingly, no STD signal was observed with MSG. In addition, changing the temperature from 7–37 °C did not produce any significant change in the STD profiles (no STD peaks) (Figure 4B), similar to CD results observed in Figure 2A over the same range of concentrations. These results indicated that the ATD domain was folded and functional and that the STD signals arise from specific interactions between neotame and the protein.

### 2.5. Monitoring Thermal Stability of Human and Mouse ATDs in the Ligand Bound and Unbound States

Monitoring the thermal stability of the tertiary structure of the human and mouse HIS-T1R2 ATD in the ligand bound and unbound states. To better understand observed changes in CD spectra of the human ATD in the presence of neotame, we applied DSC to compare the thermal stability of the tertiary structure of the human and the mouse His-T1R2 ATD constructs in the ligand-bound and unbound states. Table 2 shows a summary of the values determined from DSC for the thermo profiles of apo and neotame bound form of human and mouse ATDs. The deconvoluted thermo profiles of the unbound proteins fit best to two-state transitions for ATDs from both species. Addition of the ligand neotame resulted in a thermal destabilization of both transitions by 2.5 and 8.5 °C, respectively, and a small loss of overall enthalpy (∆H) for the h-T1R2 ATD but not when the mouse counterpart was examined.

These results are in agreement with observed loss of alpha helical structure observed in the CD studies and suggest that this α-helix loss upon binding to neotame may be correlated with an overall thermal destabilization of the protein-ligand complex. In contrast, when the mouse counterpart of the T1R2 ATD was used with neotame, only small decreases in T_m_ were observed (<1 °C for each transition), and the overall ∆H increased for the protein in the presence of ligand (Table 2), indicating no ligand interaction as expected. These results demonstrate the usefulness of the DSC method for discerning the thermal stability of the species-specific T1R2 ATD protein in the presence and absence of ligand. 

## 3. Discussion

Currently, sweet receptor extracellular domains are difficult to study due to their large size and unfavorable properties. The sweet receptor is heterodimeric consisting of T1R2/T1R3 subunits. The receptor contains large extracellular domains. These large domains mediate the interaction of ATDs with small natural and synthetic sweeteners. T1R2 ATD contains proposed binding pockets for dipeptides (e.g., neotame, aspartame), proteins (brazzein, monellin), and small natural and artificial sweeteners (Figure 1A). In order to understand the molecular mechanism by which the sweet taste response is generated, we have used a set of methodologies to measure binding events that correlate with previous sweet taste assays. Earlier work [8] described the ATD region of T1R3 as a soluble, folded and functional protein. We extend the work now to include the equivalent portion of human T1R2 and compared it with mouse T1R2, which is known to have species-specific binding properties. We have used the recombinant protein that contains the ATD region of human T1R2 as a model system to target small molecule ligands. Furthermore, we demonstrate that the ATD region of human or mouse T1R2 is capable of dimerization on its own, as monitored by gel filtration chromatography, and is able to bind to sweet ligands with micromolar binding constants (Table 1). These results are in agreement with previous studies of the full heterodimeric receptor (T1R2/T1R3) [3,4,12]. 

The combination of biophysical tools (CD, STD-NMR and DSC) provide a roadmap for studying the binding mechanism of small molecules, prior to downstream events that lead to a sweet taste response. In addition, we show that MSG, a ligand specific to the umami receptor (T1R1), does not bind to ATDs of T1R2. Similarly, our tests show that the SUMO protein tag is not involved in the binding process and could be used as a negative control (data not shown). CD spectroscopy shows a loss of secondary structure in the human ATD upon its binding to neotame, while DSC shows destabilization of the protein-ligand complex. It is likely that this thermal destabilization is correlated with the loss of α-helical content upon binding that was observed by CD. CD measurements in the near-UV region of the spectrum are clearly valuable to obtain both qualitative and quantitative information. As such, we used this sensitive tool to calculate dissociation constants for a number of sweet ligands in the presence of ATD h-T1R2 protein (Table 1). Furthermore, the use of the STD-NMR technique allowed direct observation of binding interaction of neotame with the extracellular domain of T1R2 subunit. Although STD-NMR could be used for determining apparent dissociation constants these measurements should be taken with care and require optimization of several experimental conditions to avoid overestimating *K*_D_ values [13]. To overcome limitations in each particular biophysical measurement, we combined these complementary molecular tools to access ligand binding interaction modes. In addition, our approaches are complementary to the existing calcium imaging assay in cells that require a fully functional heterodimeric receptor (T1R2/T1R3) to report on the receptor response to sweeteners. In addition, our platform provides in depth analysis of new sweeteners either alone or in competition and/or inhibition studies to fully understand the structure/functional role of ATD region of T1R2 that transpires in the initial binding event prior to sweet taste response. 

Recently, Dong and colleagues [14] have used fullerenol as a model for the sweet-taste receptor to investigate the binding affinities of structural enantiomers of sweet-taste ligands. Their basic findings demonstrated a correlation between sweet intensity and binding energy. Our methodologies would be well suited to confirm these results using the actual protein-binding interface. In addition, these results would give a more detailed description of the events that lead to either a productive or destructive binding event. 

In addition, other laboratories [12] have used taste receptor molecules to study the binding of various small sweet molecules. In particular, Masuda et al. studied the cellular responses to sweet taste stimulus in transiently transfected cells and cellular responses to sweet taste stimulus by monitoring calcium flux. The calcium-imaging assay is a downstream event that yields very little information on the molecular actions that involve a sweet-taste response in the receptor itself. Our model system for studying these interactions, in contrast, can lead to uncovering the seminal details that occur in a productive sweet taste response.

In conclusion, we suggest the application of these complementary methods to a wide range of research in other GPCR receptors. Just to name a few, we can use our expression and purification system on the equivalent ATD regions of T1R3 and T1R1, and use site-directed mutagenesis to elucidate important amino acids in these binding events. We can also study a much wider range of sweet tasting ligands, as well as those that are inhibitors (such as phenoxy herbicides) or enhancers of sweet taste response. 

## 4. Materials and Methods

### 4.1. Production and Purification of Mouse and Human T1R2 ATD Constructs

The ATD region of the protein was cloned into a 8xHis tag vector with either a TEV cleavage site or in a SUMO vector [10]. The human constructs ranged from residues Asn24-Met494 for human (ATD-hT1R2) in the SUMO fusion, or Ser25-Thr489 in the 8xHis-Tag. The mouse 8xHis-Tev-ATD-mT1R2 construct ranged from residues Gly2-Pro466. DNA coding for the amino terminal ligand binding domain of the sweet taste receptor from ATDs was expressed as inclusion bodies in *Escherichia coli* BL21-CodonPlus(DE3)-RIPL cells grown at 37 °C in 1 L of LB medium. The purification follows a procedure that has been previously described, with some modifications [9,10]. The inclusion bodies were solubilized in 6 M guanidinium chloride and refolded by dialysis against a buffer containing 50 mM Tris-acetate (pH 8.0), 50 mM KCl, 2 mM Zwittergent 3-14 and 2 mM DTT. The amino terminal domain (ATD) was purified to homogeneity on a Superdex 200 prep grade FPLC column and analyzed by 12% SDS-PAGE. The protein concentration was determined by the Bradford method using bovine serum albumin as the standard [15]. The yield from the 1 L of culture media was 5–10 mg of final protein.

### 4.2. Saturation Transfer Difference Spectroscopy (STD-NMR)

The ligand binding activity of ATD-T1R2 for all ligands was confirmed with a NMR-STD binding assay as previously described [11,16]. Aliquots of the pure labeled SUMO-T1R2 protein were incubated with desired titrating ligands at 0.5–20 fold molar excess and concentrated to a final concentration of ~0.05 mM in 10 mM phosphate buffer (pH 7.4) containing 150 mM NaCl, 2.7 mM KCl, 5 mM DTT, 5x protease inhibitor (Roche) and 0.05% NaN_3_. Monosodium glutamate (MSG, non-sweet molecule) was used as a negative control. NMR data were collected on a VNMRS spectrometer (Varian, Palo Alto, CA, USA) operating at 800 MHz and equipped with a cryogenic probe. STD-NMR data collection and analysis was carried out as previously described [13].

### 4.3. Circular Dichroism Spectroscopy (CD) 

CD spectra were recorded at 25 °C on an Model 202SF circular dichroism spectrometer (Aviv, Lakewood, NY, USA) equipped with a Peltier temperature control. Samples were added to a 0.01 cm path length quartz cuvette with a concentration of the ATD (from human and mouse) of about 0.3 mg/mL (4.5 μM) in 10 mM Tris-HCl, 150 mM NaCl, 10% glycerol, pH 7.4. Data was collected every 1 nm with an averaging time of 5 s. The spectral bandwidth was 1 nm. Spectra were corrected for buffer and ligands contributions and converted to mean ellipticity in deg cm^2^·dmol^−1^. The content of α-helix was computed using the deconvolution program from Chen et al. [16]. Titrating concentrations for ligands were from zero (no ligand present) to maximum 3 mM (above saturating concentrations) in the presence of fixed concentration (4.5 μM) of the ATD protein. The *K*_D_ was calculated by analyzing CD data at a fixed wavelength versus ligand concentration using a nonlinear regression method (see Table 1) [17]. MSG was used as a negative control.

### 4.4. Intrinsic Fluorescence Spectroscopy (Fl)

Samples were prepared in the same way as for the CD experiments. Experiments were performed at 25 °C on Carey Eclipse instrument (Agilent, Santa Clara, CA, USA). Spectra were subtracted from the buffer ± ligand backgrounds for presentation. The curves at 340 nm were the best fits of the data obtained by a regression method using Microsoft Excel software (Microsoft, Redmont, WA, USA). 

### 4.5. Differential Scanning Calorimetry (DSC)

DSC was performed using a VP-DSC microcalorimeter (Microcal, Malvern, United Kingdom). Samples of His-T1R2 ATD at about 20 mM were dialyzed against 4 × 1 L of 10 mM Tris-HCl, 150 mM NaCl, 10% glycerol, pH 7.4 in the absence or presence of 0.2 mM ligand for 16 h at 4° C prior to use. Buffer without protein was hermetically sealed in the reference and sample compartments and repeated thermograms were generated from 10–95 °C and at 1 °C/min. After confirming that the repeated buffer/buffer thermoprofiles were identical, the protein sample was exchanged in the sample compartment and scanned against the reference buffer. Data was analyzed using the Microcal software package. The thermograms were background subtracted, normalized for concentration to ∆C_p_ (mcal·deg^−1^) and baselines established on the pre- and post-transitional data. The T_m_ (°C) and ∆H (kcal·mol^−1^) were determined from the corrected curves using non-2-state transitional curve fitting.

## 5. Conclusions

We have described a protein purification system by which we can both produce and purify the ATD region of the human and mouse T1R2. We have described complementary experimental strategies for studying the conformational and binding variations that occur in the ATD domain of T1R2 of the human and mouse sweet receptor. This domain contains the majority of the proposed binding pockets for many known small molecule sweeteners that upon binding lead to the productive execution of downstream sweet taste response. These biophysical methods along with STD-NMR provide important analytical tools to quantify and map ligand binding sites of natural and synthetic sweeteners for this complex GPCR, which are crucial for understanding upstream binding events leading to a successful downstream sweet taste response.

## Figures and Tables

**Table 1:** Measured *K*_D_ values by CD for the human T1R2 ATD.

Ligand	*K*_D_ Values
Acetasulfame	100 μM
Aspartame	>300 μM
Cyclamate	ND
Neotame	50 μM
Saccharin	>1 mM
Sucrose	40 μM
Sucralose	40 μM
d-Tryptophan	500 μM
MSG	ND

**Table 2:** Summary of DSC measurements for the apo and neotame bound states of human and mouse ATD proteins.

**Parameters**	**His-hT1R2 ATD − Neotame**	**His-hT1R2 ATD + Neotame**
T_m_1	52.19 ± 0.22 °C	49.64 ± 0.14 °C
∆H1	14,980 ± 2170 cal/mole	17,770 ± 2510 cal/mole
T_m_2	62.60 ± 0.91 °C	54.09 ± 0.41 °C
∆H2	22,030 ± 2300 cal/mole	18,500 ± 2620 cal/mole
	**His-mT1R2 ATD − Neotame**	**His-mT1R2 ATD + Neotame**
T_m_1	54.88 ± 0.11 °C	54.20 ± 0.15 °C
∆H1	20,390 ± 1260 cal/mole	50,010 ± 2910 cal/mole
T_m_2	62.49 ± 0.32 °C	61.78 ± 0.89 °C
∆H2	15,130 ± 1170 cal/mole	25,300 ± 3280 cal/mole
